# p53 Phosphomimetics Preserve Transient Secondary Structure but Reduce Binding to Mdm2 and MdmX

**DOI:** 10.3390/biom9030083

**Published:** 2019-03-02

**Authors:** Robin Levy, Emily Gregory, Wade Borcherds, Gary Daughdrill

**Affiliations:** 1 Department of Cell Biology, Microbiology, and Molecular Biology, University of South Florida, Tampa, FL 33620, USA; robinlevy@mail.usf.edu (R.L.); egregorylott@mail.usf.edu (E.G.); wborcher@mail.usf.edu (W.B.); 2 Center for Drug Discovery and Innovation, University of South Florida, Tampa, FL 33612, USA

**Keywords:** tumor protein p53, mouse double minute 2, mouse double minute 4, Kinase-inducible domain interacting domain, phosphorylation, phosphomimetics, nuclear magnetic resonance, intrinsically disordered proteins, transient secondary structure

## Abstract

The disordered p53 transactivation domain (p53TAD) contains specific levels of transient helical secondary structure that are necessary for its binding to the negative regulators, mouse double minute 2 (Mdm2) and MdmX. The interactions of p53 with Mdm2 and MdmX are also modulated by posttranslational modifications (PTMs) of p53TAD including phosphorylation at S15, T18 and S20 that inhibits p53-Mdm2 binding. It is unclear whether the levels of transient secondary structure in p53TAD are changed by phosphorylation or other PTMs. We used phosphomimetic mutants to determine if adding a negative charge at positions 15 and 18 has any effect on the transient secondary structure of p53TAD and protein-protein binding. Using a combination of biophysical and structural methods, we investigated the effects of single and multisite phosphomimetics on the transient secondary structure of p53TAD and its interaction with Mdm2, MdmX, and the KIX domain. The phosphomimetics reduced Mdm2 and MdmX binding affinity by 3–5-fold, but resulted in minimal changes in transient secondary structure, suggesting that the destabilizing effect of phosphorylation on the p53TAD-Mdm2 interaction is primarily electrostatic. Phosphomimetics had no effect on the p53-KIX interaction, suggesting that increased binding of phosphorylated p53 to KIX may be influenced by decreased competition with its negative regulators.

## 1. Introduction

Loss of function mutations in the p53 pathway frequently arise during cancer development [1,2,3]. Approximately half of all human tumors express p53 mutants with reduced DNA-binding affinity which reduces or eliminates transactivation [3,4]. The tumor suppressor protein p53 is a well-known intrinsically disordered protein (IDP) whose disorder is a major component of its functionality [5,6]. An IDP is a protein that lacks a fixed or ordered structure. IDPs are structurally very different from ordered proteins and tend to have distinct properties in terms of function, sequence, interactions, evolution and regulation [7,8,9]. Many IDPs like p53 form transient secondary structures and undergo coupled folding and binding when they are bound to their targets [10,11,12]. Intrinsically disordered proteins cover a range of different states from fully unstructured to partially structured [8,13,14]. Modulation of the degree of transient secondary structure affects p53’s interactions with its binding partners [15]. Posttranslational modifications (PTMs) also regulate the activity of IDPs [16,17]. In normal cells, p53 is present at low concentrations due to its interaction with the E3 ubiquitin ligase Mdm2; however, under stress conditions, p53 is phosphorylated, leading to its dissociation from Mdm2, migration to the nucleus, and the transcriptional activation of its target genes [18]. Cells with mutant or deleted p53 are unable to respond to stress appropriately, and this leads to mutations and the development of cancer [19].

Mouse double minute 2 (Mdm2), also known as E3 ubiquitin-protein ligase Mdm2 proto-oncogene, and its homolog Mmd4, also known as MdmX, are negative regulators of p53 [20,21,22]. Mdm2 is overexpressed in several human tumor types, such as soft tissue sarcomas as well as breast tumors [23]. p53 levels are suppressed by the Mdm2/MdmX heterodimer which promotes the polyubiquitination of p53 leading to its degradation [24,25]. Mouse double minute 2 and p53 are involved in an auto-regulatory feed-back loop where p53 stimulates the expression of Mdm2; Mdm2, in turn, inhibits p53 activity because it stimulates its degradation [26,27]. Even though MdmX lacks ubiquitin E3 ligase activity it is able to directly bind to and inhibit p53 activity independently of Mdm2 [24,28,29]. Cellular stress such as DNA damage activates kinases that phosphorylate p53 at residues S15, T18 and S20, which stabilize and activate p53 by inhibiting Mdm2 binding [30,31,32]. In vitro, p53 phosphorylation affects its interaction with Mdm2, where phosphorylation of S15 and S20 residues individually result in a 2 and 1.5-fold reduction, respectively [33]. Phosphorylation of T18 leads to a 19–22-fold reduction in binding affinity of p53TAD to Mdm2, and one study has described an equivalent effect of phosphorylated T18 on Mdm2 and MdmX binding to p53 [10,33,34]. Dually phosphorylated p53 at S15/T18 results in a binding reduction equivalent to that of T18 alone, suggesting that T18 phosphorylation is the driver of reduced binding affinity of p53 to Mdm2, though the T18 site of p53 cannot be phosphorylated until the S15 site is phosphorylated first [30,33,35,36,37,38]. The phosphorylation of T18creates additional charge-charge repulsion, creating an energetically unfavorable environment for p53 and Mdm2 binding, however, the contribution of phosphorylated p53’s structural changes to Mdm2 binding has not been assessed [30,35,36,37,38].

Phosphorylation of p53TAD upon cellular stress leads to increased transcription of its target genes and increased association with its coactivator, CREB binding protein (CBP)/p300 [39]. The CBP protein is a transcriptional coactivator and histone acetyltransferase that facilitates transcription initiation of p53 target genes and stabilizes p53 by acetylating its lysines that would otherwise be ubiquitinated by Mdm2 [40,41]. It contains four domains capable of binding p53TAD, and it has been proposed that all four domains may bind tetrameric p53 in the nucleus to facilitate transcription initiation [42]. Whereas Mdm2 and MdmX interact with p53’s TAD1 region, which spans approximately residues 1–40, the KIX, TAZ1, TAZ2, and IBiD domains of CBP interacts with both TAD1 and TAD2 of p53, approximately residues 41–60 [10]. Thus, CBP competes with Mdm2 and MdmX for binding to p53, though it has also been shown that CBP and Mdm2 may form a ternary complex with p53 in vitro [10]. Phosphorylation of the TAD1 region of p53 increases binding affinity with the KIX, TAZ1, and TAZ2 domains of CBP, though possibly by different mechanisms [33,43]. In this study we focus on the kinase-inducible domain interacting domain (KIX), for which the bound state of p53 has not been determined. Phosphorylation of S15 or T18 is reported to result in a 1.7–4-fold increase in binding affinity with KIX, but an increase of 3–11-fold for the same residues when binding to TAZ2. S15/T18 phosphorylation, however, has been reported to result in 16 and 8-fold changes in binding affinity for KIX and TAZ2, respectively [43]. Likewise, where binding of p53 to the KIX domain is controlled by a combination of conformational selection and electrostatic attraction, for example, TAZ2 interaction with the phosphorylated T18 is likely driven by electrostatic attraction [44,45,46].

During phosphorylation the phosphate group contains a double negative charge that affects protein conformation mainly due to the electrostatic effects that occur between the phosphate and surrounding charged atoms of the protein [47,48]. These conformational changes can be local and/or long-range, affect protein-protein interactions, and increase or decrease levels of disorder [48]. Posttranslational modifications-mediated folding of the IDP 4E-BP2 allows it to regulate translation initiation [17]. Multisite phosphorylation stabilizes 4E-BP2 and decreases affinity to its binding partner elF4E by a factor of 4000 compared to single-site phosphorylation which only decreases affinity by 100-fold [17]. Phosphorylation of T51 conforms PAGE4 into a more compact structure that still maintains a flexible state for long range interactions. Phosphorylation of PAGE4 also increases c-Jun transactivation but decreases the affinity of PAGE4 to c-Jun, which is believed to occur due to the compact structure of PAGE4 [49]. This attenuation of binding due to phosphorylation is common between many IDPs, which are known to form transient secondary structures and undergo coupled folding and binding when they are bound to their targets [9,10,11]. The first transactivation domain of p53 forms a short helix when bound to Mdm2 and MdmX anchored via the hydrophobic residues F19, W23, L26, and TAD2 forms a short helix when bound to the TAZ2 domain of CBP anchored around the hydrophobic residues I50, W53 and F54 [43,50]. Electrostatic interactions control the stability of the helix [51]. Such helices will have a macroscopic helical dipole with a partial positive charge at the N-terminus and a partial negative charge at the C-terminus, which could stabilize the helix dipole [52,53].

To study the effects of phosphorylation on protein structure and function phosphomimetic mutations have been used extensively. Phosphomimetic mutations are amino acid substitutions (Ser/Thr to Asp or Glu and Tyr to Glu) that mimic the effect of a phosphorylated residue [54,55,56]. There are no natural amino acid side chains that provide the combination of negative charge with a tetrahedral center. However, there are numerous studies showing partial phenotypes when aspartic acid is substituted for phospho-serine or glutamic acid is substituted for phospho-threonine [39,57]. In our phosphomimic mutants, the TAD1 helix, which corresponds to residues 19–25, has one or two additional negative charges added towards the N-terminus. The addition of negative charge might be thought to stabilize the helix as seen between antiparallel alpha helices where the close proximity of opposing charges stabilizes each [53]. However, computational studies have predicted that p53 T18 phosphorylation would destabilize the helix by causing a long-range interaction with the K24 residue of p53, interfering with the D21 interaction with K24 [50]. Relatedly, phosphorylation of S20 is predicted to increase helical propensity by stabilizing the D21-K24 interaction [51].

In this present study, we obtain insights into the role of phosphorylation in modulating interactions of p53 with Mdm2, MdmX, and CBP/KIX and the effect of phosphorylation on transient secondary structure. We assessed the effects of single- and double-site phosphomimetic mutations of p53TAD upon binding to the N-terminal domain of Mdm2, MdmX, and the KIX domain of CBP. Due to the location of S15 and T18 in a region of p53TAD containing transient helical secondary structure and the importance of their phosphorylation for regulation, we engineered p53TAD phosphomimetics, S15D and S15D/T18E, to determine if the phosphomimicry of p53TAD at these sites affects protein-protein binding and changes the levels of transient helical secondary structure. Our data shows that multisite phosphomimicry reduces the binding affinity of p53TAD to Mdm2 and MdmX. In contrast to earlier published results on p53 phosphorylation, single and multisite phosphomimetics of p53TAD have equivalent, small effects on binding with KIX [33,43,46]. We observe little, if any, change to the transient secondary structure of p53TAD.

## 2. Materials and Methods

### 2.1. Purification of Mdm2, MdmX, KIX, and Labeled p53TAD Constructs

All cell growth experiments were performed in M9 media. In order to make ^15^N-labeled or ^15^N- and ^13^C-labeled samples 1 g/L of ^15^N-labeled ammonium chloride and/or 0.2% (*w*/*v*) ^13^C-labeled glucose were added in the place of nitrogen and carbon sources (Cambridge Isotopes, Andover, MA, USA).

Samples of human Mdm2 and MdmX, corresponding to residues 17–125 and 23–111, respectively, were expressed using pGEX-6p-2 vectors in BL21 (DE3) *Escherichia coli* cells grown in M9 medium. These cultures were induced at an OD_600_ of 0.6 with 1 mM Isopropyl-β-d-thiogalactopyranoside and grown for 18 h at 15 °C. Cultures were centrifuged at 11,000× *g* and frozen at −80 °C. Pellets were resuspended in glutathione S-transferase (GST) binding buffer (25 mM Tris Base, 25 mM Tris-HCl, 300 mM NaCl, 2.5 mM ethylenediaminetetraacetic acid (EDTA), 0.02% NaN_3_, 2 mM dithiothreitol (DTT), pH 7.4) containing protease inhibitors (Thermo Fisher, Rockford, IL, USA) per 2 L of culture and lysed with a French Press pressure cell using a minimum pressure of 20,000 pounds per square inch (psi). The lysate was centrifuged at 38,720× *g* for 1 h. The supernatant was filtered and then applied to a column containing 25 mL Glutathione Sepharose 4 Fast Flow resin. Protein fractions were eluted with three column volumes of 25 mM Tris Base, 25 mM Tris-HCl, 300 mM NaCl, 2.5 mM EDTA, 0.02% NaN_3_, 2 mM DTT, pH 7.4 and 10 mM reduced glutathione. Fractions were analyzed using polyacrylamide gel electrophoresis (PAGE) and those fractions containing the protein were combined and dialyzed into 25 mM Tris Base, 25 mM Tris-HCl, 300 mM NaCl, 2.5 mM EDTA, 0.02% NaN_3_, 2 mM DTT, pH 7.4, and the GST tag was cleaved using a 1:100 ratio of Human Rhinovirus 3C (HRV3C) protease. Samples were then applied to a column containing 25 mL Glutathione Sepharose 4 Fast Flow resin. Fractions were analyzed using PAGE and those fractions containing the protein were combined and dialyzed into gel filtration buffer (50 mM NaH_2_PO_4_, 300 mM NaCl, 1 mM EDTA and 0.02% NaN_3_, pH 7.0) containing 2 mM DTT. The constructs were then loaded onto a GE HiLoad 16/60 Superdex 75 column. The column was equilibrated and the protein eluted with gel filtration buffer at a flow rate of 1.5 mL/min. Protein purity was verified using PAGE analysis.

The KIX (586–672) construct was expressed as N-terminal fusions with a 7-His tag. The plasmid was transformed into BL21 (DE3) *Escherichia coli* cells from New England Biolabs (Ipswich, MA, USA) for expression using the heat-shock method then plated on agar plates that contained kanamycin for expression. Single colonies from this transformation were used to inoculate 50 mL cultures of M9 media that were grown overnight. The overnight cultures were then re-inoculated into 2 L of M9 media at an OD600 of 0.04. These cultures were induced at an OD600 of 0.6 with 1 mM Isopropyl-β-d-thiogalactopyranoside and grown for 22 h at 15 °C. Cultures were centrifuged at 11.000× *g* and frozen at −80 °C. After expression, pellet was suspended in 25 mL of lysis buffer (50 mM NaH_2_PO_4_, 300 mM NaCl, 10 mM Imidazole, 0.02% NaN_3_, pH 8.0) containing protease inhibitors (Thermo Fisher, Rockford, IL, USA) per 2 L of culture and lysed with a French Press pressure cell using a minimum pressure of 20,000 psi. The soluble fraction was isolated by centrifugation at 38,720 g for 1 h. The supernatant was filtered and added to a column containing 30 mL of Ni-NTA Superflow resin (Qiagen, Hilden, Germany). All buffers used on the NiNTA column were run at a flow rate of 3 mL/min. The column was washed with two column volumes of lysis buffer and the p53 eluted with three column volumes of elution buffer (50 mM NaH_2_PO_4_, 300 mM NaCl, 250 mM imidazole, 0.02% NaN_3_, pH 8.0). Fractions were analyzed using PAGE and those fractions containing the protein were combined and dialyzed into gel filtration buffer (50 mM NaH_2_PO_4_, 300 mM NaCl, 1 mM EDTA and 0.02% NaN_3_, pH 7.0) using 3500 Da MWCO dialysis tubing (FisherBrand, Pittsburg, PA, USA). The p53 protein was then concentrated in an Amicon Ultra-15 3K centrifugal filter device and the HIS-tag was removed by cleaving for 3 h at room temperature for the p53TAD WT (p53TAD) and overnight at room temperature for the other constructs with the Sigma-Aldrich Thrombin CleanCleave Kit (RECOMT) (St. Louis, MO, USA). The completion of the cleavage reaction was verified using PAGE. The cleaved p53 constructs were dialyzed in lysis buffer and then further purified by chromatography on Ni-NTA resin. Fractions were analyzed using PAGE and those fractions containing the protein were combined and dialyzed into gel filtration buffer and the constructs were then loaded onto a GE HiLoad 16/60 Superdex 75 column. The column was equilibrated and the protein eluted with gel filtration buffer at a flow rate of 1.5 mL/min. Protein purity was verified using PAGE analysis.

Constructs for S15D (residues 1–73), and S15D/T18E (residues 1–73) were generated by Polymerase chain reaction (PCR) site-directed mutagenesis QuickChange II (Agilent Technologies, Santa Clara, CA, USA) using the following primers for S15D d(GTCGAGCCCCCTCTGGATCAGGAAACATTTTC) and d(GAAAATGTTTCCTGATCCAGAGGGGGCTCGAC) and for S15D/T18E d(CGAGCCCCCTCTGGATCAGGAAGAATTTTCAGACCTATGG) and d(CCATAGGTCTGAAAATTCTTCCTGATCCAGAGGGGGCTCG). All p53TAD constructs were expressed as N-terminal fusions with a 7-HIS tag. The samples were then cleaved with Fisher Bioreagents OPTIMIZED (Rockford, IL, USA) Dpn1, then were transformed into XL1-Blue cells from Agilent Technologies using agar plates that contained kanamycin. The plasmids from the bacterial colonies were isolated using the Thermo Scientific GeneJET Plasmid Miniprep kit. The plasmid samples were sequenced at Eurofins Genomics and then transformed into BL21 (DE3) *Escherichia coli* cells from New England Biolabs using the heat-shock method then plated on agar plates that contained kanamycin for expression. Single colonies from this transformation were used to inoculate 60 mL cultures of M9 media that were grown overnight. The overnight cultures were then re-inoculated into 2 L of M9 media at an OD_600_ of 0.04. These cultures were induced at an OD_600_ of 0.6 with 1 mM Isopropyl-β-d-thiogalactopyranoside and grown for 5 h at 37 °C. Cultures were centrifuged at 11,000× *g* and frozen at −80°C. After expression, pellets containing unlabeled and double-labeled (^15^N, ^13^C) p53TAD peptides were suspended in 25 mL of lysis buffer (50 mM NaH_2_PO_4_, 300 mM NaCl, 10 mM Imidazole, 0.02% NaN_3_, pH 8.0) containing protease inhibitors (Sigma Aldrich) per 2 L of culture and lysed with a French Press pressure cell using a minimum pressure of 20,000 psi. The soluble fraction was isolated by centrifugation at 38,720 g for 1 h. The supernatant was filtered and added to a column containing 30 mL of Ni-NTA Superflow resin (Qiagen, Hilden, Germany). All buffers used on the NiNTA column were run at a flow rate of 3 mL/min. The column was washed with two column volumes of lysis buffer and the p53 eluted with three column volumes of elution buffer (50 mM NaH_2_PO_4_, 300 mM NaCl, 250 mM imidazole, 0.02% NaN_3_, pH 8.0). Fractions were analyzed using PAGE and those fractions containing the protein were combined and dialyzed into gel filtration buffer (50 mM NaH_2_PO_4_, 300 mM NaCl, 1 mM EDTA and 0.02% NaN_3_, pH 7.0) using 3500Da MWCO dialysis tubing (FisherBrand, Pittsburg, PA, USA). The p53 protein was then concentrated in an Amicon Ultra-15 3K centrifugal filter device and the HIS-tag was removed by cleaving for 4 h at room temperature for the p53TAD and overnight at room temperature for the other constructs with the Sigma-Aldrich Thrombin CleanCleave Kit (RECOMT) (St. Louis, MO, USA). The completion of the cleavage reaction was verified using PAGE. The cleaved p53 constructs were dialyzed in lysis buffer and then further purified by chromatography on Ni-NTA resin. Fractions were analyzed using PAGE and those fractions containing the protein were combined and dialyzed into gel filtration buffer and the constructs were then loaded onto a GE HiLoad 16/60 Superdex 75 column. The column was equilibrated and the protein eluted with gel filtration buffer at a flow rate of 1.5 mL/min. Protein purity was verified using PAGE analysis.

### 2.2. Isothermal Titration Calorimetry

Isothermal titration calorimetry (ITC) experiments were performed using a GE MicroCal VP-ITC instrument. Proteins were dialyzed against 50 mM NaH_2_PO_4_, 150 mM NaCl, 1 mM EDTA, 0.02% NaN_3_, 8 mM β-mercaptoethanol, pH 6.8. Binding experiments involving KIX were dialyzed against 50 mM Tris, 50 mM NaCl, pH 7.0. Experiments were performed at 25 °C. The typical concentration of p53 constructs (syringe) ranged from 50–500 μM and for Mdm2, MdmX and KIX (cell) 5–50 μM. Peptide concentrations were determined by absorbance at 280 nm. A typical ITC experiment consisted of one injection of 5 μL, followed by 29 injections of 10 μL up to a 2.5-fold molar excess of titrant. Data were analyzed with the Origin70 ITC software from MicroCal. Averages and standard deviations from three different ITC experiments are shown. Integrated ITC data were fit with single-site binding models and the stoichiometry ranged from 0.8 to 1.2. Errors in K_d_ were calculated from triplicate measurements.

### 2.3. Nuclear Magnetic Resonance Spectroscopy

Nuclear magnetic resonance (NMR) experiments for p53TAD and the phosphomimetics were performed using uniformly ^15^N- and ^13^C-labeled samples at 50 μM, at 25 °C on a Varian VNMRS 800 MHz spectrometer equipped with a triple-resonance pulse field Z-axis gradient cold probe. To make the amide ^1^H and ^15^N as well as ^13^Cα, ^13^Cβ, and ^13^CO resonance assignments, sensitivity-enhanced ^1^H–^15^N heteronuclear single quantum coherence (HSQC) and three-dimensional HNCACB and HNCO experiments were performed on the uniformly ^15^N- and ^13^C-labeled samples in 90% H_2_O/8% D_2_O, 50 mM NaH_2_PO_4_, 50 mM NaCl, 1 mM EDTA, and 0.02% NaN_3_, pH 6.8. For the HNCO the Varian VNMRS 600 MHz spectrometer with a triple resonance pulse field Z-axis gradient cold probe was used. The sweep widths were 9689.9 (t_3_) Hz × 3770.1 (t_2_) Hz × 1944.5 (t_1_) Hz, and complex data points were 1024 (t_3_) Hz × 64 (t_2_) Hz × 32 (t_1_) Hz. For p53TAD the HSQC and the HNCACB were performed on the 600 MHz spectrometer. The sweep widths and complex points for the HSQC were 7225.4 (t_2_) Hz × 1500 (t_1_) Hz and 1024 (t_2_) Hz × 128 (t_1_) Hz, respectively. The HNCACB experiment, data were acquired in the ^1^H, ^13^C, and ^15^N dimensions using 7225.4 (t_3_) Hz × 12063.8 (t_2_) Hz × 1500 (t_1_) Hz sweep widths and 1024 (t_3_) Hz × 128 (t_2_) Hz × 32 (t_1_) Hz complex data points. For S15D the HSQC was performed on the 600 MHz spectrometer and the HNCACB was performed on the 800 MHz spectrometer. The sweep widths and complex points for the HSQC were 7266 (t_2_) Hz × 1943.2 (t_1_) Hz and 1024 (t_2_) Hz × 128 (t_1_) Hz, respectively. The HNCACB experiment, data were acquired in the ^1^H, ^13^C, and ^15^N dimensions using 9689.9 (t_3_) Hz × 14074.9 (t_2_) Hz × 1944.3 (t_1_) Hz sweep widths and 1024 (t_3_) Hz × 128 (t_2_) Hz × 32 (t_1_) Hz complex data points. For S15D/T18E the HSQC and the HNCACB were performed on the 800 MHz spectrometer. The sweep widths and complex points for the HSQC were 9689.9 (t_2_) Hz × 1944.4 (t_1_) Hz and 1024 (t_2_) Hz × 128 (t_1_) Hz, respectively. The HNCACB experiment, data were acquired in the ^1^H, ^13^C, and ^15^N dimensions using 9689.9 (t_3_) Hz × 14074.9 (t_2_) Hz × 1944.3 (t_1_) Hz sweep widths and 1024 (t_3_) Hz × 128 (t_2_) Hz × 32 (t_1_) Hz complex data points. All NMR spectra were processed with NMRFxProcessor and analyzed using NMRView J software (Standford, CA, USA) [58,59]. Secondary chemical shift values were calculated by subtracting the residue specific random coil chemical shifts in the prediction of temperature, neighbor and pH-corrected chemical shifts for intrinsically disordered proteins (POTENCI) from the measured chemical shifts [60]. Secondary structure populations were calculated with δ2D using the measured proton, nitrogen, and α, β, and carbonyl carbon chemical shifts [61]. The overall helicity was calculated as the mean of the per residue δ2D helical population estimates.

## 3. Results and Discussion

**Figure 1:**
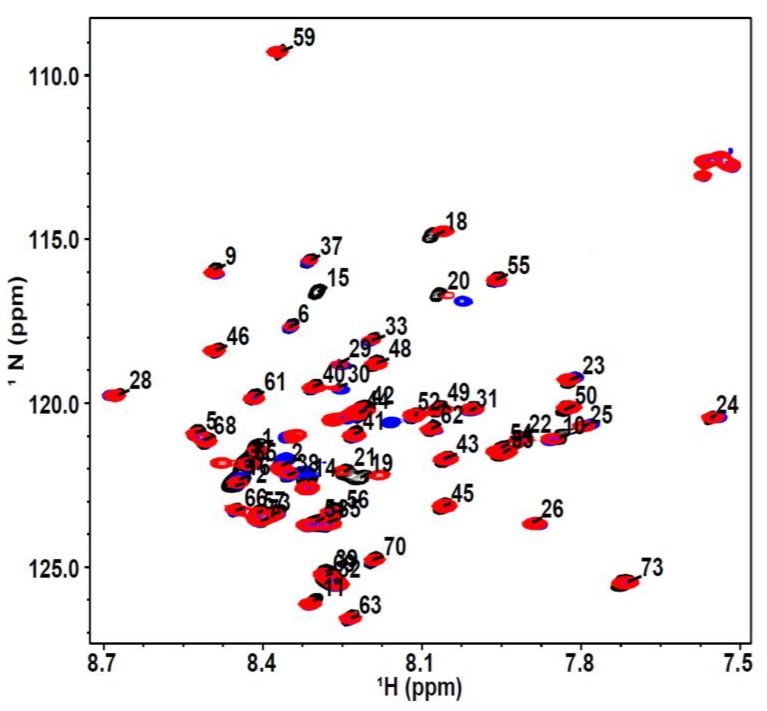
p53TAD and phosphomimics. ^1^H-^15^N HSQC spectra overlay of ^15^N-labeled p53TAD (black), ^15^N-labeled S15D mutant (red), ^15^N-labeled S15D/T18E mutant (blue).

We attempted in vitro phosphorylation experiments with NMR labeled p53TAD using DNA-PK and CK1γ2 kinases to determine changes to transient secondary structure but were unable to get 100% phosphorylation at either S15 or T18 compared to that of previous studies (Figure S1) [62]. Therefore, we chose to use phosphomimetic mutations. The phosphorylation of p53 makes it more negatively charged. In studying protein phosphoreguation, it has become common to mutate phosphorylation sites to phosphomimetic residues to attempt to study the constitutively phosphorylated state of the protein [33,55,56,63,64,65,66,67]. We designed p53TAD phosphomimetics (residues 1–73) by mutating S15 to Asp and S15/T18 to Asp/Glu, which will be referred to as S15D and S15D/T18E, respectively. We used NMR spectroscopy to measure any changes in the transient secondary structure of p53TAD wild type (p53TAD) and mutants. An overlay of the ^1^H-^15^N heteronuclear single quantum coherence (HSQC) spectra of p53TAD and the phosphomimetics is shown in Figure 1. The labeled peaks show the resonance assignments of p53TAD residues (black peaks). There is hardly any shift in the majority of the residues for the S15D (red peaks) and S15D/T18E (blue peaks) mutants compared to p53TAD. We do see a significant shift at residues that are close to the mutated sites of S15 and T18 suggesting that any structural effects from the mutation(s) will be local.

**Figure 2:**
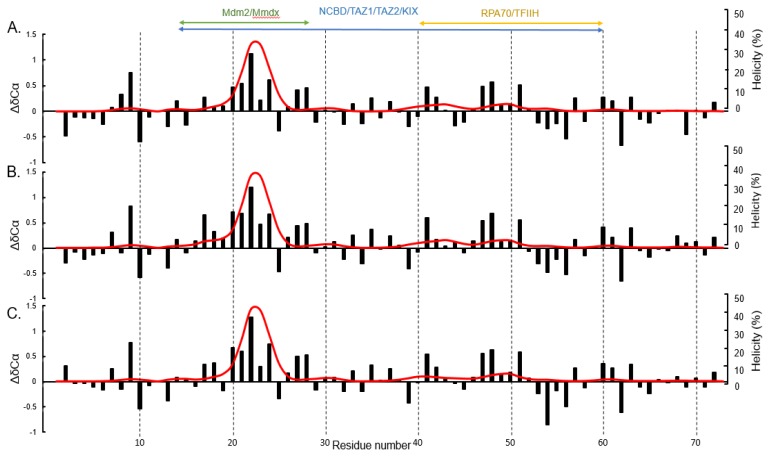
Residue specific secondary structure of p53TAD and phosphomimetics. Secondary chemical shift plots and δ2D plot for p53TAD and phosphomimetics determined from nuclear magnetic resonance (NMR) spectroscopy. (**A**) p53TAD (**B**) S15D (**C**) S15D/T18E. α-carbon secondary chemical shift (ΔδCα, black bars) and helical δ2D plots (red line) for the p53TAD and phosphomimetics as determined by NMR spectroscopy. Colored bars indicate binding sites for respective protein partners. The α-carbon chemical shifts for p53TAD was collected on a 600 MHz NMR at a digital resolution of 0.31 ppm. The alpha carbon chemical shifts for S15D and S15D/T18E was collected on an 800 MHz NMR at a digital resolution of 0.27 ppm.

Secondary chemical shift values were calculated using the prediction of temperature, neighbor and pH-corrected chemical shifts for intrinsically disordered proteins (POTENCI) software (Figure 2). This software calculates residue specific random coil chemical shifts from an amino acid sequence and these values are subtracted from the NMR measured chemical shift values to give the corrected secondary chemical shift values [60]. Positive alpha carbon secondary chemical shifts are indicative of alpha helix formation [68,69]. All of the measured chemical shifts (NH, N, CA, CB, and CO) were used to calculate the distribution of transient secondary structure using the δ2D software [61]. The negative charge produced during phosphorylation affects protein conformation mainly due to the electrostatic effects and these changes can be local and long-range, effect protein-protein interactions, and increase or decrease levels of disorder [47,48]. A short helix compromising residues 19–25 has one or two additional negative charges added towards the N-terminus in our phosphomimetic mutants which might stabilize the helix due to the close proximity of opposing charges. We observed small differences in the transient helical secondary structure between p53TAD, S15D and S15D/T18E (Figure 2). However, of the changes that were present most were within the Mdm2/MdmX binding site. There was a slight increase in helicity for the mutants, with p53TAD having 36.4% helicity at its highest point and S15D and S15D/T18E having 39.8% and 39.9% helicity at their highest points, respectively, as indicated by the δ2d plots (Figure 2 red line). The reported accuracy of δ2D is 2%. The changes in the secondary chemical shifts, though minimal, were also observed within the Mdm2/MdmX binding site (Figure 2 black bars).

**Figure 3:**
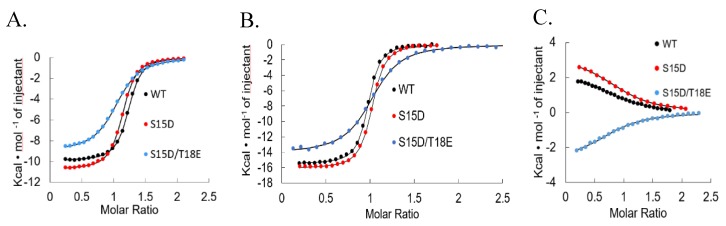
Binding Isotherms of Mdm2, MdmX and KIX with p53TAD and mutants. Isothermal calorimetry titrations of (**A**). Mdm2 and p53TAD and mutants (**B**). MdmX and p53TAD and mutants and (**C**). KIX and p53TAD and mutants. WT: wild type.

Next, we used isothermal titration calorimetry (ITC) to determine the effect of the phosphomimetic mutations on p53TAD binding to Mdm2 (residues 17–125), MdmX (residues 23–111), excluding the N-terminal “lid” and KIX (residues 586–672) (Figure 3) [70,71,72]. Compared to qualitative methods of measuring protein-protein binding (e.g., immunoprecipitation, western blot, GST pull-down) ITC is a widely used technique for quantitative studies of an extensive variety of biomolecular interactions [73,74,75,76,77]. It is mostly used to observe the binding between molecules like protein and DNA by measuring the binding affinity, enthalpy and stoichiometry of interacting molecules [73]. Isothermal titration calorimetry measures the heat that is either expelled or consumed by the interaction of the molecules present and modern ITC instruments make it possible to measure the differences in heat as small as 0.1 μcal (0.4 μJ) [78]. It can simultaneously determine multiple binding parameters in a single experiment and does not require the modification of binding partners with fluorescent tags or through immobilization; ITC measures the affinity of binding partners in their native states. Isothermal titration calorimetry experiments were performed by titrating the p53TAD phosphomimetics into Mdm2, MdmX, and KIX. The ITC experiments were performed in triplicate and the values averaged (Table 1). Data were analyzed with the Origin70 ITC software from MicroCal and the integrated ITC data were fit with single-site binding models and the stoichiometry ranged from 0.8 to 1.2. A standard deviation was calculated for K_d_ using data from triplicate measurements. Our results showed that p53TAD and S15D bound Mdm2/MdmX with similar affinities, whereas S15D/T18E displayed a 2.5–4.5-fold reduction with Mdm2 and a 5-fold reduction with MdmX (Figure 3A,B). Binding affinity of p53 to KIX was similar between p53TAD and all the phosphomimetics. Binding of p53TAD to KIX was endothermic with similar values for S15D (Figure 3C and Table 1). However, S15D/T18E was exothermic (Figure 3C and Table 1). Interestingly, the phosphomimetics had no effect on the transient helical secondary structure of p53TAD. Taken together, the results argue that binding affinity between phosphorylated and unphosphorylated p53 to Mdm2/MdmX is primarily controlled by electrostatics and the binding to KIX is unchanged.

## 4. Conclusions

Using phosphomimetic mutations we investigated the effect of phosphorylation on the binding of p53 with Mdm2, MdmX, and CBP/KIX and the transient secondary structure of p53TAD. By increasing negative charge of neighboring residues at the positive end (N-terminus) of the helix formed by p53 in the bound state with Mdm2, we expected a stabilization of the helical dipole in accordance with what is seen in antiparallel helix interactions [53]. Conversely, simulations suggest that phosphorylation of T18 has a destabilizing effect on the helix in the bound state [30]. Our results show that neither an increase nor decrease in transient helicity occurs for S15D or S15D/T18E. 

Binding affinities of the p53TAD phosphomimetics for the Mdm2, MdmX and KIX were determined using ITC (Figure 3). For both Mdm2 and MdmX, S15D showed similar binding to p53TAD. S15D/T18E showed a decrease in binding to Mdm2 and MdmX with binding being 4.5 times and 5 times weaker than p53TAD, respectively (Table 1). Many studies have shown that phosphorylation of S15 and T18 play a critical role in preventing the interaction with Mdm2 [32,35,79]. There has been some disagreement regarding the impact of phosphorylation on binding; there is a good consistency for unphosphorylated peptides (residues 10–57) binding to Mdm2 but there is some variation in the binding affinity of phosphorylated peptides that does not appear to correlate with different techniques or sizes of the peptides being used [10,43,80,81]. Though we do not see much structural change with the phosphomimetics, we do see binding results consistent with other studies that phosphorylated p53 at the same residues. Previous studies showed a 10–20-fold reduction in binding of p53 to Mdm2 due to p53 phosphorylation as compared to our results where we see a 3–5-fold reduction [10,43,80,81,82]. The results of our binding experiments with phosphomimetics are consistent with previous findings suggesting that the phosphorylation of T18 is the driving force for inhibiting Mdm2/MdmX binding [30,50,80,81,82,83]. Furthermore, there has not yet been a quantitative study of the effect of p53 phosphorylation on MdmX binding. Our results suggest that p53 phosphorylation may in some cases have an equivalent effect on MdmX as on Mdm2.

Binding affinity of p53 to KIX was not significantly altered between p53TAD and the phosphomimetics. We found p53TAD binds to KIX with a K_d_ of 11 μM, similar to values found in previous studies [10,33,46]. In contrast to previous studies on p53 phosphorylation, however, these phosphomimetic mutations increased binding affinity for KIX by only 1.3-fold. Phosphorylation of p53 at S15 has been shown to result in a 1.7–4-fold increase and S15/T18 phosphorylation results in a 16-fold increase in binding affinity to KIX in vitro [10,33,43,84]. The increase in binding affinity of phosphorylated p53 to the domains of CBP has been attributed to an increase in electrostatic attraction independent of site-specific affinity; however, it is unclear if this trend applies to KIX, which has been suggested to have a relatively weak response to phosphorylation of p53 compared to other CBP/p300 domains [43]. Our results show that an increase in negative charge of p53TAD alone is not sufficient to significantly increase binding affinity for KIX [33]. Instead, it seems that the increase in binding affinity to KIX by p53 phosphorylation may occur by way of a structural change that is not fully replicated in the phosphomimetics produced here. We postulate that the phosphomimetics of p53 created here represent an intermediate phenotype between that of phosphorylated and unphosphorylated p53 and may be useful for future cell and molecular biology studies.

## Figures and Tables

**Table 1:** Isothermal titration calorimetry (ITC) values for interactions between Mdm2, MdmX and KIX with p53TAD and mutants.

		p53TAD	S15D	S15D/T18E
**Mdm2**	K_d_ (nM)	219.5 ± 0.012	392.5 ± 0.015	1.001 ± 0.016
	ΔG (Kcal/mol)	−9.1	−8.75 ± 0.071	−8.2
	ΔH (Kcal/mol)	−9.721 ± 0.202	−11.335 ± 0.827	−8.173 ± 1.203
	TΔS (Kcaal/mol/deg)	−0.634 ± 0.236	−2.499 ± 0.977	−0.964 ± 0.188
**MdmX**	K_d_ (nM)	29 ± 0.004	30 ± 0.002	108 ± 12.5
	ΔG (Kcal/mol)	−10.3 ± 0.100	−10.27 ± 0.058	−9.5 ± 0.07
	ΔH (Kcal/mol)	−16.260 ± 0.913	−16.99 ± 1.405	−14.27 ± 0.19
	TΔS (Kcal/mol/deg)	−5.960 ± 0.988	−6.794 ± 1.55	−4.75 ± 0.16
**KIX**	K_d_ (nM)	11,000 ± 2.700	8200 ± 1870	8380 ± 969
	ΔG (Kcal/mol)	−6.77 ± 0.157	−6.95 ± 0.127	−6.92 ± 0.056
	ΔH (Kcal/mol)	2.606 ± 0.308	2.926 ± 0.416	−2.822 ± 0.079
	TΔS (Kcal/mol/deg)	9.377 ±0.165	9.874 ± 0.308	3.963 ± 0.270

## Supplementary Materials

The following are available online at https://www.mdpi.com/2218-273X/9/3/83/s1, Figure S1: Partial phosphorylation yields no chemical shifts in the primary MDM2 binding region.
