# Supplementary material for: p53 Phosphomimetics Preserve Transient Secondary Structure but Reduce Binding to Mdm2 and MdmX
Source: Biomolecules. 2019 Mar 2;9(3):83. doi: 10.3390/biom9030083 (PMC6468375; doi:10.3390/biom9030083)
Supplement: Supplementary file 1 [file biomolecules-09-00083-s001.pdf]

Supplementary:

p53 Phosphomimetics Preserve Transient Secondary Structure but Reduce Binding to Mdm2 and MdmX

Robin Levy [1,2], Emily Gregory [1,2], Wade Borcherds [1,2] and Gary Daughdrill [1,2,*]

[1] Department of Cell Biology, Microbiology, and Molecular Biology, University of South Florida, Tampa, FL 33620, USA; robinlevy@mail.usf.edu (R.L.); egregorylott@mail.usf.edu (E.G.); wborcher@mail.usf.edu (W.B.)

[2] Center for Drug Discovery and Innovation, University of South Florida, Tampa, FL 33612, USA

* Correspondence: gdaughdrill@usf.edu; Tel.: +1-813-974-2503

Academic Editors: Prakash Kulkarni and Vladimir N. Uversky
Received: 23 January 2019; Accepted: 28 February 2019; Published: 2 March 2019

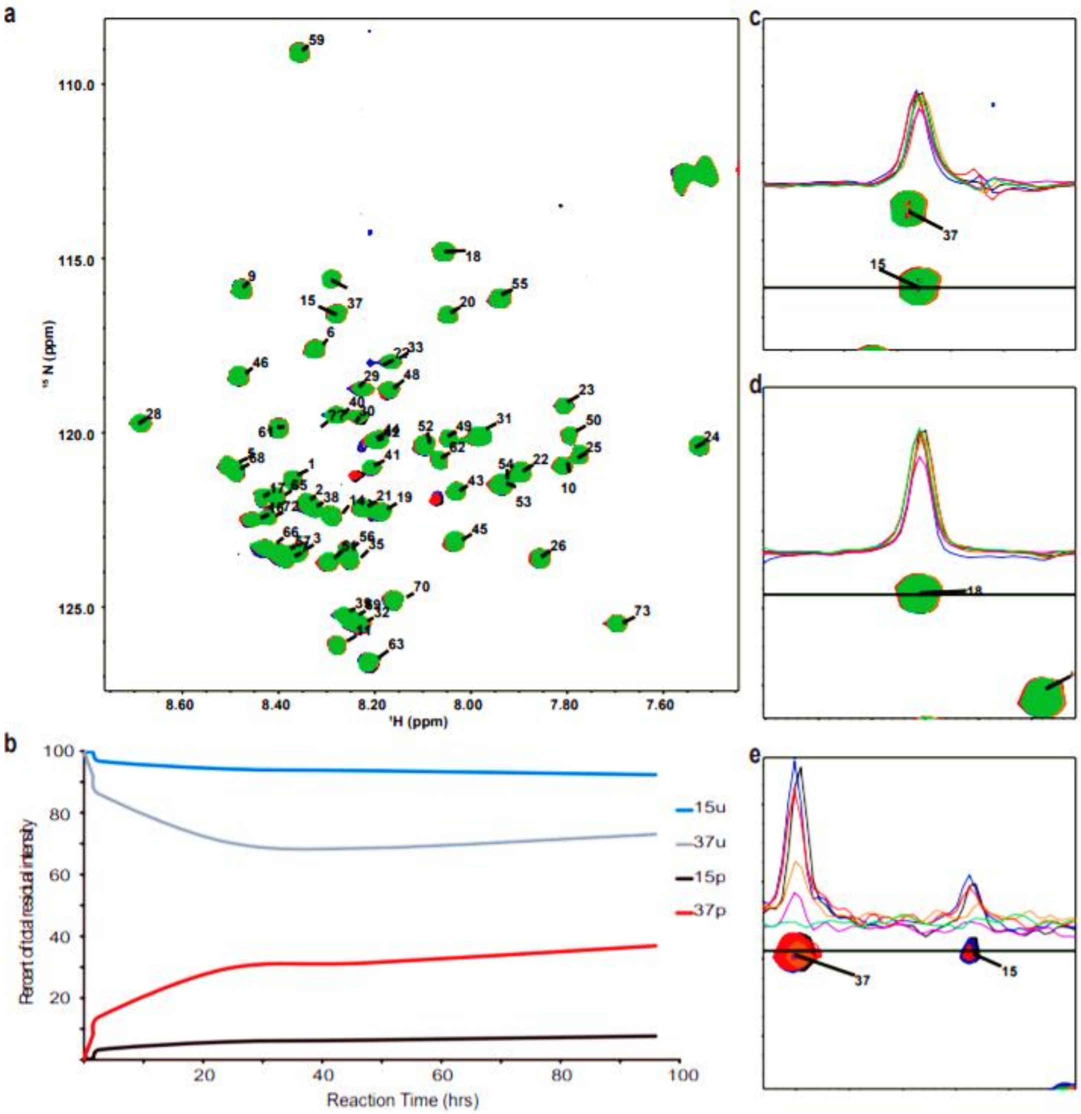

Figure S1. Partial phosphorylation yields no chemical shifts in the primary Mdm2 binding region. (a) The overlaid spectra of p53WT TAD1-73 unphosphorylated (green) overlaid with 1.5 h (purple), 2.5 hour (orange), 24 h, (red) 48 h (blue) and 96 h (black) incubations with DNA-PK. (b) shows the percent of total residual intensity for the phosphorylated (p) peak, or the unphosphorylated (u) peak over the sum of the two peaks as a function of incubation time. (c) shows the zoomed in overlay of unphosphorylated peaks for S15 and S37 with a 1d slice of the 1H for S15. (d) The zoomed in overlay of T18 with a 1d 1H Slice. (e) The zoomed in overlay of the phosphorylated peaks of S37 and S15 that occur downfield of the main spectra with a 1d 1H slice running through both.
